# Health Outcomes at 1 Year After Thermal Ablation for Cervical Precancer Among Human Papillomavirus– and Visual Inspection With Acetic Acid–Positive Women in Honduras

**DOI:** 10.1200/GO.20.00400

**Published:** 2020-10-19

**Authors:** Rose C. Slavkovsky, Pooja Bansil, Manuel A. Sandoval, Jacqueline Figueroa, Doris M. Rodriguez, Jose Saul Lobo, Jose A. Jeronimo, Silvia de Sanjosé

**Affiliations:** ^1^ PATH, Seattle, WA; ^2^ Asociación Hondureña de Planificación de Familia (ASHONPLAFA), Tegucigalpa, Honduras; ^3^ Secretaría de Salud de Honduras, Tegucigalpa, Honduras; ^4^ National Cancer Institute, Bethesda, MD

## Abstract

**PURPOSE:**

This study aims to assess the detection of cervical intraepithelial lesions grades 2 and 3 (CIN2-3) at 1 year after treatment with thermal ablation among human papillomavirus (HPV)–positive and visual inspection with acetic acid (VIA)–positive women.

**METHODS:**

All women screened and triaged for cervical cancer at four government health facilities in Honduras who were eligible for ablative treatment were enrolled and treated with thermal ablation. Women with confirmed CIN2-3 and a subset of women with CIN1/normal diagnoses at baseline were evaluated at 12 months. Follow-up procedures included HPV testing (*care*HPV), VIA, directed biopsy (if VIA-positive), and Papanicolaou test (if HPV positive, VIA negative). Outcomes at 1 year included histologic or cytologic assessment of CIN lesions among those with any abnormal test.

**RESULTS:**

Among the 319 women treated with thermal ablation, baseline histologic diagnoses were available for 317. Two (0.6%) had invasive cancer, 36 (11.4%) had CIN3, 40 (12.6%) had CIN2, and 239 (75.4%) had CIN1/normal histology. Among the 127 women eligible for follow-up, 118 (92.9%) completed all study procedures at 1 year. Overall, 98 (83.1%) had no evidence of CIN2-3 or persistent low-grade infection, 13 (11.2%) had CIN1/atypical squamous cells of undetermined significance, six (5.1%) had CIN2/high-grade squamous intraepithelial lesion, and 1 (0.8%) had a persistent CIN3. No adverse events associated with thermal ablation at 1 year were registered.

**CONCLUSION:**

A high proportion of women had no evidence of CIN2-3 at 1 year after thermal ablation treatment. Thermal ablation is an alternative to cryotherapy that may facilitate greater treatment coverage and prevent unnecessary deaths from cervical cancer.

## INTRODUCTION

As the fourth most common cause of cancer incidence and mortality among women internationally, cervical cancer is a global public health problem.^1^ An estimated 570,000 new cases developed in 2018, with 87% of these in low- and middle-income countries (LMICs). In Honduras, cervical cancer is the second leading cause of cancer incidence and the leading cause of cancer mortality among women, with an estimated 800 new cases and 480 deaths annually.^2^

**CONTEXT**

**Key Objective**
To evaluate treatment impact of cervical thermal ablation after close follow-up for 12 months.
**Knowledge Generated**
The majority of women showed no signs of cervical disease at 12 months after treatment with thermal ablation. Thermal ablation was evaluated as a highly acceptable approach.
**Relevance**
Treatment of cervical intraepithelial lesions with thermal ablation is easy to manage and accessible to low-resource settings.

Prevention of cervical cancer is achieved through vaccination to avert infection with oncogenic genotypes of human papillomavirus (HPV) and through screening, diagnosis, and treatment of cervical precancer.^3-5^ Although secondary prevention can reduce cases of invasive cervical cancer, it requires that screen-positive women receive timely and effective precancer treatment.^5^ As part of the global call for action on cervical cancer elimination, the WHO target for screening programs is to provide treatment to 90% of women with cervical disease.^6^ Standard treatment recommendations for cervical precancer in LMICs has been cryotherapy, the application of a cooled probe tip to the cervix to ablate lesions.^7^ As documented extensively, cryotherapy presents several logistical challenges for health systems that ultimately preclude eligible, screen-positive women from receiving treatment.^8-10^

The WHO issued new guidelines in 2019 recommending thermal ablation for treatment of precancerous lesions.^11^ Thermal ablation consists of applying a probe tip heated to between 100°C and 120°C to the cervix to destroy precancerous lesions; the goal is to treat the whole transformation zone (TZ), with and without a lesion,^12^ with the aim to remove lesions of an average depth of 5 mm from the surface of the cervical epithelium.^13^ Although data on successful treatment after thermal ablation are limited to primarily observational studies,^14^ treatment success is expected to depend on HPV status and full removal of the TZ.^11^ Studies have shown rates of cure after thermal ablation to be equivalent to or better than those achieved via cryotherapy,^11^ and the procedure has been shown to be safe and well-tolerated by women.^8,15-17^

Newer thermal ablation devices are battery powered, handheld, and portable, facilitating treatment in primary health centers and through mobile outreach. Despite these advances, there is limited published evidence on clinical outcomes after thermal ablation from LMICs and, specifically, from the Latin America region. This study aims to assess the level of detection of cervical intraepithelial neoplasia (CIN) lesions and HPV infection among women 1 year after treatment with thermal ablation in Honduras.

## METHODS

Study sites consisted of four government health facilities in the metropolitan region of Francisco Morazán in Honduras, one of four regions in the country where the Secretary of Health recently implemented HPV testing for cervical cancer screening.^18^ As described previously, the study enrolled 319 women.^15^ Briefly, participants with a positive result for both HPV and visual inspection with acetic acid (VIA) were invited and assessed for eligibility. Enrolled and consented participants underwent a confirmatory VIA evaluation and had one or more directed biopsies before receiving thermal ablation treatment. The Liger Medical HTU-110 Thermocoagulator (Liger/Cure Medical, Lehi, UT) device was selected because it was the only handheld battery-powered thermal ablation device commercially available when the study was designed in 2016. Baseline biopsies were classified as CIN 1, 2, or 3, or as invasive cancer. The biopsies were diagnosed by a local expert pathologist, and a sample was externally reviewed for quality control by a second expert pathologist in Costa Rica. Discrepant results were reevaluated by both pathologists. Women with a histologic detection of invasive cancer exited the study and were referred for adequate care.

Results from the 1-month follow-up visit (visit 2) to assess short-term safety and acceptability of outcomes have been reported.^15^ Women were scheduled for 1-year follow-up if they had histologically confirmed CIN2-3. A sample of women—those with normal/CIN1 diagnosis during the final 2 months of recruitment—were also included in the study follow-up for quality control purposes. For the remaining women with a normal/CIN1 diagnosis who exited the study according to the protocol, study staff carried out a chart review to determine the number of women who returned for routine VIA follow-up per national guidelines and their VIA results at 1 year; no HPV tests were available to women attending routine follow-up. Within the study, women underwent up to three follow-up visits at 1 year (visits 3-5). Visit 3 consisted of provider- or self-collected HPV sample collection. At visit 4 after HPV results were available, the provider performed VIA in all women and obtained one or more directed biopsies from any visible lesion among VIA-positive women, regardless of the HPV result. A Papanicolaou (Pap) test was taken at visit 4 among women with a positive HPV result but a negative VIA evaluation. Women with both negative HPV and VIA evaluations exited the study. Women who underwent biopsies or Pap returned for their results within 1 month (visit 5), when they received their results before exiting the study. Women with a positive Pap or biopsy result were managed according to the lesion severity. On exiting the study, women with negative results were recommended for routine follow-up at the primary health level. All women received a small stipend for each follow-up visit attended to cover the cost of transportation to and from the health facility.

Investigators obtained institutional review board approvals for the original study protocol and subsequent amendments from PATH’s Research Ethics Committee and the Comité de Ética en Investigación Biomédica of Universidad Nacional Autónoma de Honduras in Tegucigalpa. The study is registered with ClinicalTrials.gov (ClinicalTrials.gov identifier: NCT03510273).

All statistical analyses were conducted in Stata 13.1 and 16 (StataCorp, College Station, TX). Descriptive statistics of the following baseline characteristics and follow-up clinical procedures and outcomes were calculated: geographic site, age, number of probe applications and type of probes used, HPV and VIA follow-up test results, number of biopsies at follow-up, and histology and cytology results at follow-up. The number and frequency of persistent infections and evidence of clinical outcomes stratified by baseline biopsy results (less than CIN2 and CIN2+) were evaluated and compared using a two-sample proportion *t* test. We used Poisson regression with robust variance to estimate the unadjusted and adjusted prevalence ratios of CIN2-3 with various factors at 1 year. VIA test results for women with normal/CIN1 diagnosis who were followed up via standard care at 1 year were used to estimate the overall treatment success rates among women who received thermal ablation treatment. We define treatment success as having no evidence of CIN2-3 at 1 year.

## RESULTS

**FIG 1:**
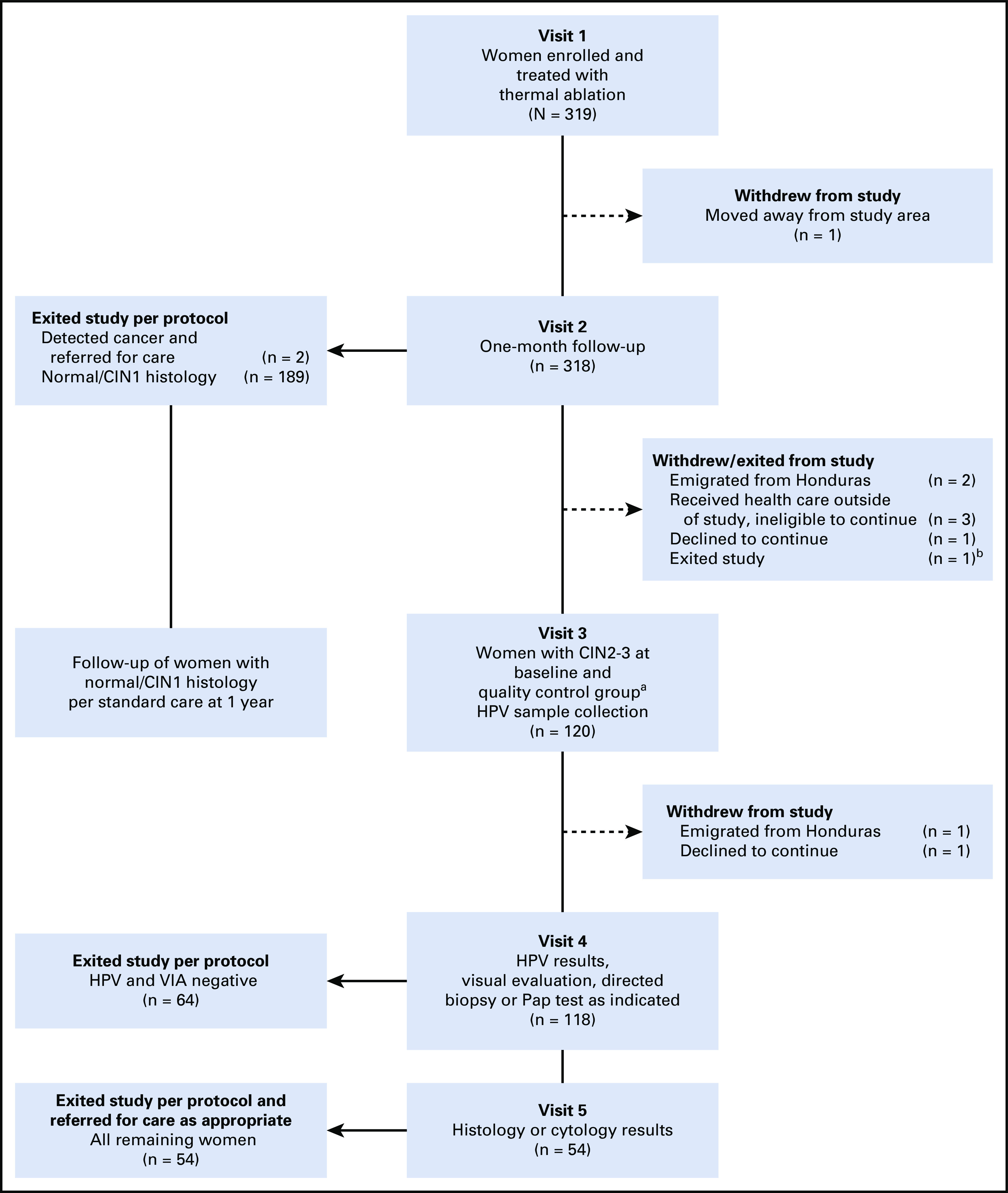
Study algorithm from entry to 1-year follow-up. (^a^) Quality control group consisted of a sample of 51 women with normal/cervical intraepithelial neoplasia 1 (CIN1) histology at baseline. (^b^) One woman exited the study with unknown histology. HPV, human papillomavirus; Pap, Papanicolaou; VIA, visual inspection with acetic acid.

Among the 319 HPV- and VIA-positive women enrolled and treated at baseline, histologic diagnoses were available for 317. Two women were identified as having invasive cancer (0.06%), 36 (11.4%) had CIN3, 40 (12.6%) had CIN2, and 239 (75.4%) had CIN1/normal histology. All 76 women with CIN2-3, 50 women with CIN1/normal histology, and one woman with no baseline biopsy result were anticipated for follow-up at 1 year. Among these 127 women, three women received health care outside the study and were ineligible to continue, three emigrated from Honduras, two declined to continue, and one woman exited the study, resulting in 118 (92.9%) women who completed all scheduled follow-up visits, as described in Figure 1. Of these, 69 (58.5%) had a CIN2-3 diagnosis at baseline and 49 (41.5%) had a negative histology for CIN2-3. At 1 year follow-up and according to the protocol, 64 women with a negative HPV and VIA evaluation exited the study after visit 4, and 54 returned for visit 5 within 1 month.

Baseline characteristics of all study participants and the subset of women who attended all follow-up visits at 1 year are shown in Table 1. Among women who completed all evaluations at 1 year, the majority were from Crucitas (73.7%), and more than a third were between 30 and 34 years of age (35.6%). Of those receiving one application, 54.2% were treated with a flat 19-mm tip. Among women who received two or more applications, 50.5% were treated with a nipple 19-mm tip.

**TABLE 1:**
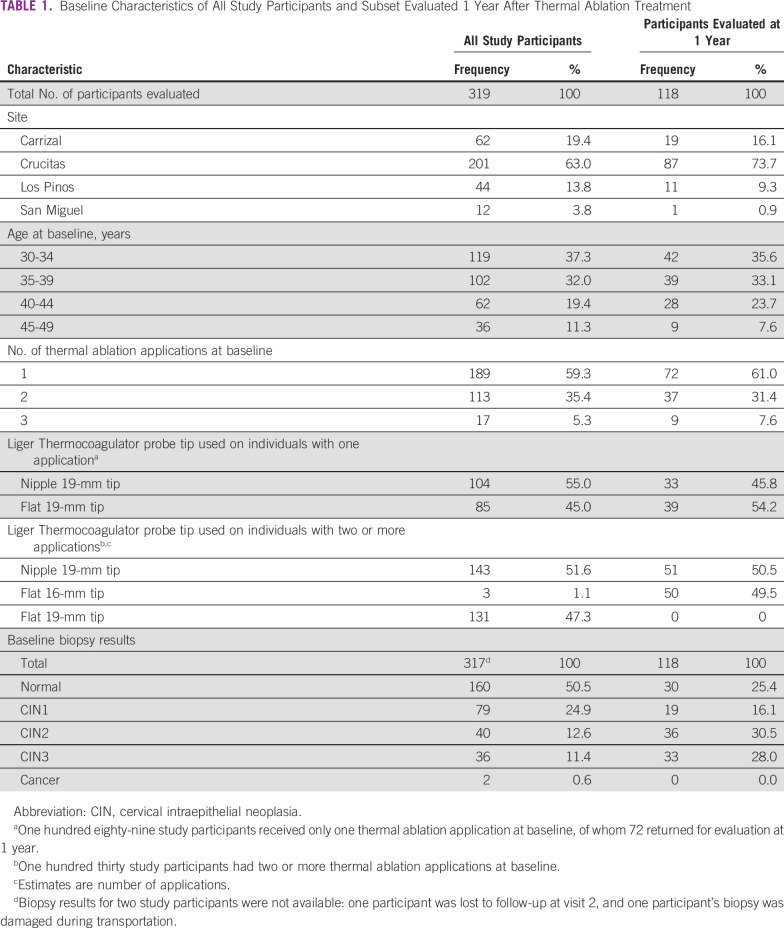
Baseline Characteristics of All Study Participants and Subset Evaluated 1 Year After Thermal Ablation Treatment

Table 2 describes the clinical results of the 118 women who completed all follow-up procedures at 1 year stratified by baseline histology. Of these, 28 (23.7%) remained HPV positive, 40 (33.9%) were VIA positive, and 14 (11.9%) remained positive for both HPV and VIA. Among the 40 women who were VIA positive, 32 (80.0%) had two or more biopsies taken (data not shown), and 34 (85.0%) had normal or CIN1 histology. Of the 14 women who were HPV positive and VIA negative, 12 (85.7%) had normal cytology, one had an atypical squamous cells of undetermined significance (ASC-US), and one had a high-grade squamous intraepithelial lesion (HSIL).

**TABLE 2:**
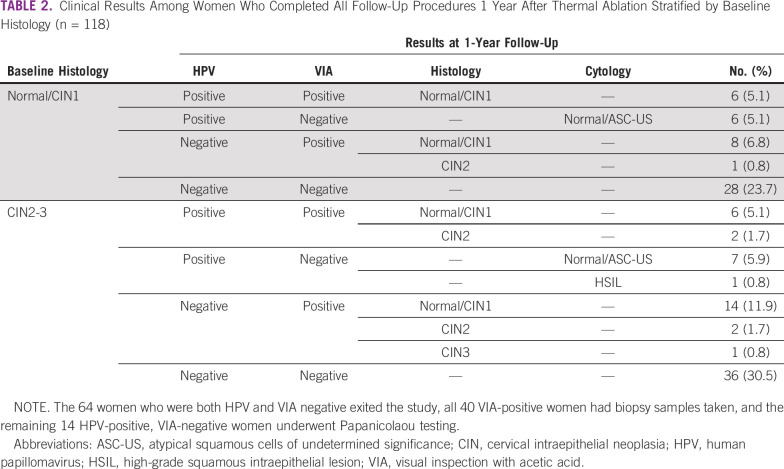
Clinical Results Among Women Who Completed All Follow-Up Procedures 1 Year After Thermal Ablation Stratified by Baseline Histology (n = 118)

Table 3 summarizes the evidence of CIN2-3 or persistent detection of HPV infection among women at 1 year. Overall, 98 (83.1%) had no evidence of CIN2-3 or infection at 1 year, 13 (11.0%) had a CIN1 or ASC-US, and seven women (5.9%) had CIN2-3 or HSIL. Among the seven women considered to have treatment failure, one had a baseline histology of CIN1 and a CIN2 at follow-up (Table 4). The remaining six had a persistent outcome of CIN2+ or HSIL, one of whom had a CIN3 diagnosis at baseline and was HPV negative at 1 year (Tables 2 and 4). Thus, out of the 69 women with CIN2-3 at baseline, only six had CIN2+ or HSIL at 1 year (87.0%). Of the 7 CIN2+ cases at 1 year, 6 were VIA positive and 3 were HPV positive. When evaluating associations between baseline characteristics and the prevalence ratio of CIN2-3 at follow-up, there were no statistically significant differences (at the *P* = .05 threshold) on the basis of study site, age, number of treatment applications, probe tip used, or number of biopsies taken at follow-up in both adjusted and unadjusted models, and there was no difference between women with less than CIN2 and CIN2-3 at baseline (data not shown).

**TABLE 3:**
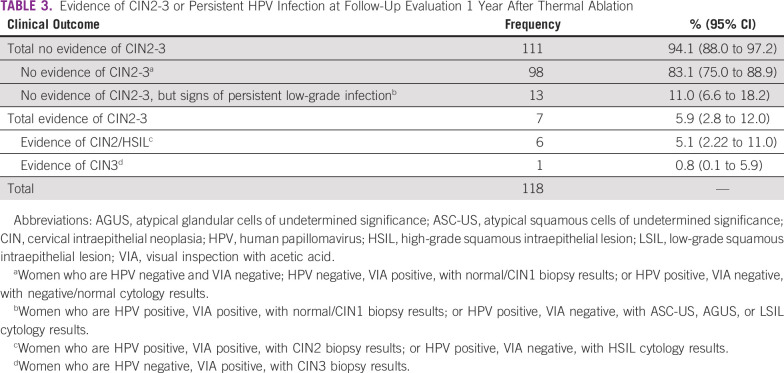
Evidence of CIN2-3 or Persistent HPV Infection at Follow-Up Evaluation 1 Year After Thermal Ablation

**TABLE 4:**
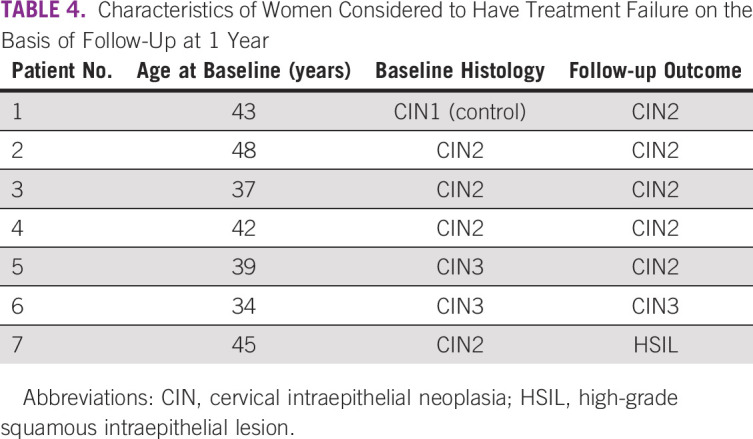
Characteristics of Women Considered to Have Treatment Failure on the Basis of Follow-Up at 1 Year

Among women with less than CIN2 histology results at baseline who were followed with routine screening (VIA) at 1 year, clinical evaluation was retrieved from 128 of 189 women (67.7%). Among these, 17 (13.3%) were VIA positive and 111 (86.7%) were VIA negative.

The 1-year outcome for all women, including those followed up in the study and as part of standard care, indicates that a majority (90.2%) had no evidence of disease, 2.8% had evidence of CIN2-3, and 12.2% showed some evidence of HPV infection.

## DISCUSSION

In this longitudinal study of women treated for cervical precancer in Honduras, we found that thermal ablation resulted in a high level of treatment success, measured at 1 year. Including women actively followed in the study and women who attended routine follow-up through the public health system, 90.2% of women positive for HPV and VIA at baseline had no evidence of CIN2-3 at 1 year, and only one was diagnosed with CIN3, the immediate precursor lesion of cervical cancer. These findings complement our previous analysis of safety and acceptability of thermal ablation in the same population, which found the procedure to be safe, accepted, and well tolerated by women.^15^

This study was embedded within the standard clinical approach of treating HPV- and VIA-positive women, substituting thermal ablation for cryotherapy. To evaluate treatment success, histology evaluation at baseline and at follow-up was considered essential; however, the baseline histology results identified 75.4% of women negative for CIN2-3. This proportion of normal results among HPV- and VIA-positive women was higher than our initial estimates and could be explained by a poor accuracy of VIA and/or poor sensitivity of one single biopsy in the majority of cases, taken without the aid of a colposcope.^11,19^ In response, we first modified the protocol to keep a sample of women with normal/CIN1 histology for complete follow-up evaluation at 1 year. This quality control group provided an estimate of whether cases of high-grade CIN were missed with the small number biopsies taken at baseline. We anticipated that at 1 year, the proportion of women with CIN2-3 in the quality control group would be less than the proportion among women with CIN2-3 at baseline. As expected, there was one diagnosis of CIN2 (2.0%) in the quality control group compared with six (8.7%) in those with CIN2-3 at baseline. Second, we updated our study procedures, instructing that two or more biopsies be taken at 1 year.

Our evaluation of treatment success among HPV- and VIA-positive women 1 year after thermal ablation also reflects the reality of screening programs in limited-resource settings, where it is rare to obtain histologic diagnosis before precancer treatment, and several countries rely solely on VIA positivity as an indication for treatment.

Our results show that one woman had a new or progressed lesion as compared with six who had persistent or progressed lesions. Although we did not have HPV type–specific data or the location of the original acetowhite lesions, the small number of abnormalities detected at follow-up indicates that thermal ablation treatment is highly effective in HPV- and VIA-positive women. In future studies, information on the site of the lesion and HPV type pre- and post-treatment could allow for distinguishing between new and persistent lesions and provide greater insight into treatment performance.

Our results indicating treatment success among 90.7% of HPV- and VIA-positive women and 82.0% among those with CIN2-3 lesions at baseline are consistent with a recent systematic review conducted by the WHO of randomized and nonrandomized studies, which found the proportion of women with no evidence of disease after thermal ablation to be 91%.^11^ In addition, our level of treatment success is higher when compared with preliminary findings from an ongoing randomized controlled trial in Zambia comparing thermal ablation, cryotherapy, and large loop excision of the transformation zone, which evaluated similar treatment outcomes at 6 months.^17^ Although there are several differences between this study and ours—including enrollment age, prevalence of HIV in the underlying population, and follow-up evaluations—in the Pinder et al^17^ study, 83% of HIV-negative women at baseline in the thermal ablation arm had no evidence of disease at 6-month follow-up, compared with our results of 90.7% treatment success at 1 year. It could be speculated that more stringent criteria for reporting a VIA-positive diagnosis in Zambia or differences in HPV test sensitivity could explain these differences.

Regarding the small number of women with CIN2-3 at 1 year in our study, it was not possible to identify which factors (eg, number of treatment applications, probe type, age) may have contributed to these outcomes. We found no association between the number of treatment applications and the prevalence of CIN2-3 at follow-up, suggesting that the number of applications provided for the 45-second cycle time was sufficient to treat the entire TZ for the majority of women.

Although we did not find evidence of an association between the type of probe used and detection of CIN2-3 at follow-up, a two-probe method (conical probe followed by flat probe) has been reported to have higher rates of cure.^11^ More research is needed to determine whether a multiple-probe method results in significantly improved outcomes.

Our study was successful in following 92.9% of participants for 1 year, and the majority (67.7%) of women who exited the study at visit 2 received standard VIA follow-up at 1 year. This demonstrates a high level of patient retention by the primary health care centers, which is essential for the effectiveness of secondary prevention programs. Increasing follow-up and treatment coverage has greater impact on reducing cervical cancer mortality than increases in screening coverage alone.^20^ Among the three women who received care outside the study after initial treatment with thermal ablation, one underwent hysterectomy and another received conization. As both were negative for CIN2+, this shows mismanagement of patients by the health care system and underscores the importance of providing adequate training and support to providers on adherence to clinical guidelines.

Our study has some limitations. HPV-positive women were recruited in an urban setting; hence, our results may not be generalizable to other, rural regions in Honduras or other low-resource settings. Women who exited the study with normal/CIN1 results at baseline and received routine follow-up were only evaluated with VIA and not HPV testing. Although there were no cases of CIN3 among women with less than CIN2 at baseline for those with a complete follow-up, we may have missed cases among VIA-positive women who were followed up via standard of care because of lack of available information. Histologic evaluation before and after thermal ablation provided a quality check on the clinical procedures but also identified the presence of false negatives and false positives in both diagnostic tests (*care*HPV, QIAGEN, Hilden, Germany; and VIA) being used. The only woman with persistent CIN3 was self-collected HPV negative and VIA positive at 1 year. This suggests that she was either healing and her HPV infection had already cleared, that her HPV viral load was below the cutoff value for the *care*HPV test, or that her HPV result was a false negative. Overall, this observation underscores the importance of adequate follow-up of women after ablative treatment of cervical precancer. Given that the sensitivity of VIA to detect CIN2-3 can be low and with low reproducibility^11^ and that many VIA-positive women in this study did not have CIN2-3 lesions, our findings indicate overtreatment. Based on low numbers, of the 7 women with CIN2+ at 1 year, VIA detected 6 and HPV detected 3. Although the US colposcopy guidelines recommend two to three biopsies per lesion,^21^ this is difficult in a primary care setting without the aid of a colposcope and where local anesthesia is not routinely used. More research is needed to provide evidence-based recommendations on use of biopsies in a primary care setting. The use of histologic assessment, even if it is only viable in a proportion of cases, provides a quality check of the whole screening approach and should be considered when implementing screen-and-treat approaches.^22^ Furthermore, taking digital images of the cervix for an algorithm-derived diagnosis after application of VIA may considerably improve the eligibility assessment for ablative treatment and increase the quality of the interventions.^23^

This study shows a high proportion of HPV- and VIA-positive women with no evidence of CIN2-3 at 1 year after treatment with thermal ablation. Thermal ablation is a validated alternative to cryotherapy that may facilitate greater treatment coverage and prevent unnecessary deaths from cervical cancer.^24^ As of 2018, Honduras updated its national guidelines to include thermal ablation, and other countries in the region are moving forward with this technology.
